# Attitudes Toward Gender-Neutral Spanish: Acceptability and Adoptability

**DOI:** 10.3389/fsoc.2021.629616

**Published:** 2021-03-15

**Authors:** Juan Eduardo Bonnin, Alejandro Anibal Coronel

**Affiliations:** ^1^ Consejo Nacional de Investigaciones Científicas y Técnicas (CONICET), Buenos Aires, Argentina; ^2^ Ciencias Políticas y Relaciones Internacionales, Grupo Independiente de Investigación MEL (GI-MEL), Córdoba, Argentina

**Keywords:** gender-neutral language, non-gendered language, inclusive language, linguistic attitudes, sociolinguistic survey, social media, Argentina

## Abstract

This article presents the results of a survey conducted in January 2020 about attitudes toward gender-neutral language in Argentina. The survey was delivered mainly through social networks to 4,205 participants, and its results help understand the complexity of the attitudes toward the phenomenon. In particular, I will argue two hypotheses: 1) that an extensive favorable attitude of acceptance toward gender-neutral language does not imply extensive willingness to use it; 2) that its use is more readily accepted and used in vocative positions, indicating that it works better as a strategic discursive option than as an ongoing linguistic change.

## Introduction

Although the phenomenon of gender-neutral language (GNL), also known as non-gendered language, gender-inclusive language or simply inclusive language, can be traced back until at least the mid-1960s, in the past few years it has gained visibility in public discourse, mass media and social media.

In English, “preferred pronouns” and “singular they” are extremely visible examples of the way in which speakers struggle to define new resources to express non-binary gender identities. Similarly, Arabic, Hebrew, German, French and Swedish, among other languages, use different alternatives: rehabilitating out-of-use variants (such as the 14th-century singular they, in English), re-functionalizing already existent forms (such as the neutral pronouns in German, or the dual in Arabic), introducing new orthographic signs (such as the asterisk “*” in French or the underscore “_” in Slovene), etc. (Berger, 2019).

In Argentina, the issue became especially visible during 2018, when a teenager’s casual use of gender-neutral morphology in a TV interview was received with angry remarks by the interviewer, the notoriously right-wing Eduardo Feinmann (Schmidt, 2019). Since then, the issue of GNL has triggered the most extreme arguments in Argentina’s public and private sphere: Is it a linguistic aberration? Should it be prohibited? Should it be mandatory? Despite the huge social repercussions of this debate, little systematic research has been done from a sociolinguistic or discourse analysis perspective.

Thus, this article presents the results of a survey conducted in January 2020 about attitudes toward GNL in Argentina. The survey was delivered through different social networks to 4,205 participants, and its results help understand the complexity of the attitudes toward the phenomenon. In particular, we will argue two hypotheses: 1) that an extensive favorable attitude of acceptance toward GNL does not imply extensive willingness to use it; 2) that its use is more readily accepted and used in vocative positions, indicating that it works better as a strategic discursive option than as an ongoing linguistic change.

## Literature Review

The emergence of gender-neutral forms in different languages around the world is a relatively new phenomenon, which encompasses new identities and social movements, notably feminisms, transgender and gender-nonconforming or non-binary groups. In English, for example, the indication of a “preferred pronoun” has become increasingly frequent as a part of basic personal information at institutions, especially universities (Parks and Straka, 2018). The adoption of singular “they” as a pronoun used as a conscious choice to reject the traditional gender binary “he” or “she” made headlines in 2016, after well-established linguistic institutions such as the American Dialect Society and the Merriam-Webster Dictionary chose singular “they” as “word of the year” 2015. This policy regarding gender and language has been quite extensive in college campuses throughout the United States: at Harvard University, for example, 1% of the 4,000 students indicated gender-neutral pronouns at the university’s registration system in 2015 (Binkey, 2015). The visibility of non-binary identities through language choices, however, has faced resistance among staff, faculty and even students (Darr and Kibbey, 2016). It also presents technical challenges in fields where identity between grammatical gender, gender identity and biological sexuality are usually taken for granted, as in laboratory tests and medical records (Imborek et al., 2017).

The use of gender-neutral forms, and even of languages with less gendered grammar, helps women to overcome stereotyped gender roles and develop as active agents in institutions; at least that is the conclusion of Brutt-Griffler and Kim (2017) in their study of Asian female international studies at US colleges. The field of social psychology has established experimentally the impact of grammatical gender in male-bias perception, within the scope of linguistic relativity studies in a number of different languages (cfr. Boroditsky, 2003; Stahlberg et al., 2007; Everett, 2011), generally classifying “gender” in dichotomic categories (Ansara and Hegarty, 2014). In other languages, such as Slovene, the use of gender-inclusive forms is widespread only in the LGBTIQ + community and some specific cultural/media outlets. Within these groups, the use of non-binary forms is remarkably consistent, even presenting some level of variation that has been interpreted as index of linguistic vitality (Popic and Gorjanc, 2018).

Despite the existing evidence of the impact of grammatical gender on the perception of reality, and the role of generic masculine in reproducing male-biases and gender stereotypes, it is often argued that introducing gender-neutral forms in a natural language is both useless (as gender inequality goes far beyond grammar) and impossible (as speakers’ resistance defies linguistic planning). However, the case of the gender-neutral pronoun “*hen*,” in Sweden, has shown that it is both possible and has active positive effects on language attitudes and behavior (Gustaffson Sendén et al., 2015).

The Swedish form is beautifully called “gender-fair language” (“könsmässigt språk”), and introduces a neutral third person pronoun, hen, as an alternative to the existing Swedish feminine (hon) and masculine (han) alternatives. After its first appearance in print in a children’s book in 2012 (a coincidence with the Spanish gender-neutral morpheme *-e*, which appeared in a The Little Prince edition of 2018), it was included in the 2015 edition of the Swedish Academy Glossary, thus acquiring semi-official status despite the heated public argument about its adoption. However, research shows that, over time, the initial resistance to its use shifted to positive attitudes and behavior (Gustaffson Sendén et al., 2015).

Within these global processes of linguistic policies and politics, I will introduce the case of Spanish in general, in Argentina, in particular.

Spanish has a binary grammar gender system, differentiating masculine and feminine. The gender of nouns agrees with determinants and adjectives, so gender is a very pervasive feature. Nouns are always assigned a gender; from a grammatical point of view, there are no gender-neutral nouns. Masculine is often marked with the suffix -o, and can be easily changed to feminine by replacing it by a, as in “*compañero/compañera*,” or by adding the suffix if the masculine form ends in consonant, as in “*doctor/doctora*.”

Masculine has an unmarked or default status for Spanish speakers from the standpoint of grammar (Real Academia Española, 2010, chapter 11), psycholinguistics (Beatty Martínez and Dussias, 2019) or sociolinguistics (Kalinowski, 2020b). From a discursive point of view, however, this unmarked status of the generic masculine has been questioned repeatedly (cfr. Chávez Fajardo, 2019). At first, feminist criticism denounced the many forms of invisibilization of women through linguistic sexism (Fletcher, 1988). In public discourse, this critique became visible in the coordination of masculine and feminine forms when addressing a heterogeneous group of people (“*estimados y estimadas*” instead of “*estimados*”). Although the Spanish Royal Academy and other conservative linguistic institutions argued against this coordination as unnecessary (for example, by evoking a principle of linguistic economy, or style), it became fairly extended in public discourse (cfr. Pérez and Moragas, 2020) and there is little room for rejection, since it conforms to the standard norm.

A second instance of linguistic activism against linguistic sexism became more visible in the late ‘90s and early ‘00s, under the form of a non-binary morphology used to refer to collectives of people which cannot be assumed to be male, female or non-binary. When considering these gender-neutral options, the first innovations consisted in replacing the binary morphemes -a and -o by -x or -@. The “@” was progressively abandoned because of its binarism (as it evokes an “a” and an “o”), while the “x” became very widespread; even in English the term “*latinx*” became popular, at least in college campuses and academic settings. Nonetheless, there is a catch in this alternative: although it works perfectly well in written texts, it cannot be pronounced. Thus, the morpheme -e was introduced in 2012 by LGBTTIQ + activists M. Wayar and Lohana Berkins as the most suitable innovation, since it is a non-binary vowel that can be used both in written and oral discourse.

The use of the morpheme -e as a gender-neutral mark has been persistently discussed, in very heated and often aggressive terms, in mass media and social media. Pérez and Moragas (2020) analyze the process of neutralization of dissident discourses in the media by de-legitimizing feminism and LGBTIQ + spokespeople. From their perspective, the main reason to delegitimize inclusive language is its anti-binarism in terms of gender identity.

There is very little research on the topic on Spanish, most of which is devoted to discourse analysis on the topic. Many universities and public offices have developed guidelines for non-sexist and inclusive language, and scholarship in general has shown a favorable attitude toward the acceptance of non-binary forms, either standard (i.e. using epicene nouns) or non-standard (-x or -e) (Romero and Funes, 2018; Kalinowski, 2019; Martínez, 2019; Sayago, 2019; Tosi, 2019; Kalinowski, 2020b). As the issue has only gained visibility in the last three years, research on the subject is still scarce and mainly theoretical (Bolívar, 2019; Gasparri, 2020; Glozman, 2020; Kalinowski, 2020b), analysis of discourses which topicalize inclusive language (Barrera Linares, 2019; Pérez and Moragas, 2020) and discursive practices in secondary education (Castillo Sánchez and Mayo, 2019; Tosi, 2019).

Research about attitudes and actual use of inclusive language is even scarcer. Tosi (2020) analyzes the presence of gender-neutral Spanish through a survey of 30 technical copyeditors from Argentina. Her survey asked whether the copyedited manuscripts used any variant of IL, finding only 10% of affirmative responses. An interesting result of the study is that many inclusive language variants that are standard–such as using epicene nouns as “*el estudiantado*” instead of gender-varying nouns like “*los estudiantes*”—are not perceived as “inclusive language”. By adopting this broader criterion, the author finds that 60% of the copyedited texts use some of these non-sexist variants.

With regard to actual use of inclusive language, Kalinowski (2020a) conducted a corpus-based study of non-binary morphology in Twitter from 2007 to 2020 in Argentina. He found that, like in Swedish, the use of non-standard non-binary forms (such as -x and-e) increased over time. Another result of this study is that the increased usage of these forms seems to be linked to political actions and legislative initiatives [such as the Gender Identity Act of 2012, or the (failed) Interruption of Pregnancy Act of 2018], which confirms the results reported by Gustaffson Sendén et al. (2015), who show that gender identity and political preferences are predictors of positive attitudes toward inclusive language. In other terms: GNL seems to be linked both to progressive ideologies and political agenda, especially with regard to feminist and sexual rights movements in Argentina.

One remarkable conclusion of Kalinowski (2019) and Kalinowski (2020b) is that non-binary non-standard forms (such as suffixes in -e and -x) cannot be described–at least for the time being–as a part of a linguistic variety, but rather, as a discursive strategy. This conclusion is drawn from: 1) a limited, but very active, number of users; 2) a very limited number of lexical forms employing -e and -x. The latter feature is very important: 72.37% of the non-standard non-binary tokens used in Twitter correspond, in fact, to only four words: *todxs/es*, *amigxs/ues*, *elxs/les*, and *chicxs/ques*.

It can thus be concluded that gender-neutral language, as it currently exists in Argentina and can be documented in social media, is a speech phenomenon, mainly a lexical one, which is limited to a handful of words: “*chiques*,” “*amigues*” or “*todes*.” Little is known, however, about speakers’ attitudes toward these forms. Firstly, linguistic attitudes are usually described in a very simplified manner, as a scale going from “positive” to “negative,” and do not distinguish, for instance, attitudes of acceptance from willingness to act. Secondly, word count does not help understand the pragmatic and syntactic aspects of speakers’ attitudes toward inclusive language. Finally, ideological motivations in favor of or against non-binary morphology are usually described only from a gender/political ideology perspective, not taking into account linguistic ideologies; i.e., the case of someone who embraces non-binary ideas about gender but still cannot accept non-standard linguistic forms.

In what follows, the survey designed to address these three aspects will be described: 1) a more complex understanding of linguistic attitude, by differentiating “acceptability” from “adoptability”; 2) a pragmatics-based perspective on -e forms, distinguishing its vocative and non-vocative uses; and 3) a more nuanced approach to inclusive language, not only as a phenomenon related to gender/political ideologies, but also to linguistic ideologies.

## Materials and Methods

### Construction and Justification of Hypothesis

Attitudes toward inclusive language have been characterized theoretically according to 2 variables: linguistic ideologies (in this case, whether a person is more or less open to change and linguistic innovation) and ideas about gender (basically, whether a speaker considers gender as a binary or non-binary category). Combined, they provide the following typology:

As can be seen in Table 1, only one out of four possible combinations is fully identified with inclusive language understood as a non-binary non-standard form. This, of course, is a typical-ideal model; in the reality of subjective attitudes in everyday communication, the situation is more complex, and often presents multiple nuances.

**TABLE 1:** Typology of linguistic attitudes toward NGL.

	Linguistic ideology resistant to change/non-standard forms	Linguistic ideology prone to change/non-standard forms
Binary ideas about gender	A. Rejection (“alumnos”)	C. Rejection (“alumnos y alumnas”)
	Generic masculine	Binary, standard
Non-binary ideas about gender	B. Rejection of -e suffix but acceptance of standard non-binary forms (such as epicene nouns as “el alumnado” instead of “les alumnes”)	D. Acceptance (“alumnes”)
	Non binary, standard	Non-binary, non-standard

These attitudes, however, cannot be understood in simplistic terms (such as categorizing them as positive/neutral/negative), because a positive judgment about a linguistic form does nor necessarily means adoption (i.e., I accept children language, but I do not use it myself). Thus, it is important to differentiate between an attitude of acceptance toward other people’s use of a linguistic form (a variable which we call “acceptability”) from an attitude of willingness to adopt such form (which we call “adoptability”).

To understand these attitudes, a short survey that received 4,205 responses on social networks, mainly Twitter, was designed. Although the sample is not representative of the Argentine population, and therefore the results cannot be generalized, the survey shows how these attitudes are related to each other. The research design of this survey was not meant to disentangle these two dimensions systematically, but only to explore relevant cases with regard to linguistic attitudes toward non-binary, non-standard linguistic forms.

In this article we will address the following hypotheses:

1. Speakers are more willing to express acceptance toward non-binary non-standard morphology than to adopt it themselves.
2. Inclusive language is more acceptable, and people are more willing to use it, in a vocative position, at the beginning of the sentence.

### Participants and Procedure

Data on attitudes toward gender in oral speech was collected by administering two questionnaires in social networks during one week in January 2020 (see Supplementary Annex S1). The rationale for using two questionnaires was to test an additional hypothesis, which was proven false, that female voices would trigger more positive attitudes toward inclusive language than would male voices. In Form 1, the first three items are uttered by a female voice, and the latter three by a male voice, while in Form 2, a male voice is heard in the same first three items, and a woman’s voice in the latter three.

A pilot test was applied to 30 individuals chosen at random with the purpose of detecting internal inconsistencies of the questionnaire, semantic incongruities of the questions and detecting difficulties in understanding the instructions as well as the proposed response categories. As a result, the “non-binary” gender category was adopted, as an emergent from the pilot. The final questionnaire is included in Supplementary Annex S1.

Both questionnaires were distributed through a single link, and then alternatively administered by redirecting to one of two Google forms with the survey. Form 1 was answered by 1959 people, and Form 2 by 2,246, totaling 4,205 cases, selected by virtual snowball sampling.^1^ The survey was accessed mainly through Twitter (56.6%), followed by WhatsApp (28.4%), Facebook (8.3%), Instagram (1.8%), and others (4.9%). As there were no statistically significant differences^2^ in any variable of either dataset, I collapsed both into one to run the analysis, thus abandoning any hypothesis about gender-of-speaker as an independent variable (see Supplementary Annex S2).

As cases were reached by convenience, this is a non-probability sample, which means that neither generalization, nor sample error, can be estimated with any degree of confidence to the entire population of Argentina. However, it can help to better understand the ways in which different variables are associated, especially with regard to the hypothesis proposed here.

### Variables

One of the main concerns of the survey was its length, because web-based surveys have high attrition rates, especially when answered in mobile phones (Hochheimer et al., 2016). Therefore, its design was very simple, while other items were postponed for future research (such as including more pragmatic alternatives, written items or Likert scales to measure different attitudes). This is one of the limitations of the study although, on the other hand, it helped to secure a greater number of valid answers and a larger sample.

Participants listened to six short audios, the first three spoken by a woman and the last three by a man, or vice versa (depending on whether it was Form 1 or 2; cfr. Supplementary Annex S1). The phrases were the following:

1. Hey, chiques, ¿quieren venir al cine esta noche?
2. Hey, chicos, ¿quieren venir al cine esta noche?
3. Hey, chicos y chicas, ¿quieren venir al cine esta noche?
4. Les dije a todes mis amigues que vinieran al cine.
5. Les dije a todos mis amigos que vinieran al cine.
6. Les dije a todos mis amigos y mis amigas que vinieran al cine.

Grammatical markings of gender are analyzed in three non-mutually exclusive forms, following the typology presented in Table 1: non-binary, non-standard “-es”; generic masculine “-os”; and binary standard “-os and -as.” Only type b (non-binary, standard) is not represented in the survey, as no convenient epicene noun was found to serve both as vocative and non-vocative with these syntactic forms.

To account for the pragmatic meaning of gender marks vocative and non-vocative positions were distinguished, as they offer unique insight into the interface between syntax and pragmatics that can be observed in very short fragments (Shormani and Quarabesh, 2018). For the case of the non-vocative position, it is a reported speech topicalizing a previous invitation, which allows for evoking the situation without actually using the vocative form.

Attitudes toward these phrases were assessed by selecting one of the following statements:

1. I find it acceptable and I would use it.
2. I find it acceptable but I would not use it.
3. I find it weird but I would use it.
4. I find it weird and I wouldn’t use it.
5. I find it unacceptable and I wouldn’t use it.
6. I find it unacceptable, but I would use it.

Each statement offers a combination of two attitudes. The first one is acceptability, assessed in three values: acceptance, weirdness and non-acceptance. “Weirdness,” as an intermediate value, was defined as a result of qualitative exploratory studies. The second attitude is adoptability, or willingness to use, as preliminary studies showed that people can accept the use of non-binary non-standard forms in other people, but might not be willing to use it themselves, which happens to be my own case, too.

Gender identification was assessed by simple choice (male, female, non-binary, I prefer not to answer) showing the following gender profile in the sample, as can be seen in Table 2.

**TABLE 2:** Distribution gender identification—total sample (absolute frequencies and %).

Gender	Frequency (n)	Percentage (%)
Female	2,884	68.6
Male	1,207	28.7
Non-binary	69	1.6
NA	45	1.1
TOTAL	4,205	100

Source: The authors.

The table shows that women are overrepresented regarding total population of Argentina, which comprises 48.6% men and 51.3% women, with no count of non-binary population as of the last census in 2010.

On the contrary, it should be stressed that, despite we cannot determine whether Non-binary participants are either over or underrepresented regarding total population, its survey percentage participation was low. Nevertheless, for the sake of statistical analysis, having *n* = 69 implies that the statistical theoretical assumptions can be fulfilled, having no negative statistical implications at all when analyzing crosstabs.^3^

In terms of location, a list of Argentine provinces was offered when asked for “place of residence”. The sample included people from all over the country, although Buenos Aires Province and Buenos Aires City are overrepresented (together they account for 75% of total answers). This fact does not allow to consider this variable as eventually explicative of results.

Respondents were also asked for level of education, in order to test the popular hypothesis that higher educational levels could be associated with a more positive attitude toward non-binary non-standard options, both in terms of acceptability and adoptability. Analysis, however, showed no significant relationship between these two variables.^4^

Finally, age was an open question, which was later clustered into seven groups: 12–18, 18–24, 25–30, 31–40, 41–50, 51–60, 60+ (age distribution shown at Table 3). For reasons of relevance, this variable will not be analyzed here, but in a future study.

**TABLE 3:** Distribution of age—total sample (absolute frequencies and %).

Age - Re-coded	Frequency (n)	Percentage (%)	Valid percentage
<18 years	74	1.8	1.8
18–24 years	776	18.5	18.5
25–30 years	786	18.7	18.7
31–40 years	1,319	31.4	31.4
41–50 years	713	17	17
51–60 years	366	8.7	8.7
> Than 60 years	168	4	4
Valid total	4,202	99.9	100
System missing	3	0.1	—
Total	4,205	100	—

Source: The authors.

## Results

Analysis shows a statistically significant association between gender and attitudes toward gender marks, especially in the case of non-binary respondents for the extreme categories (accept and use, and reject and not use).

How do attitudes toward different forms in different positions correlate to each other? Table 4 shows how attitudes toward the six phrases proposed in the survey correlate to each other, independently of the attitude in itself.

**TABLE 4:** Correlation Matrix between attitudes toward phrases.

			Phrase 1	Phrase 2	Phrase 3	Phrase 4	Phrase 5	Phrase 6
Spearman’s rho	Phrase 1	Correlation Coeff	1	−0.179^a^	0.056^a^	0.761^a^	−0.190^a^	0.069^a^
		Sig. (bilateral)		0	0	0	0	0
		N	4,205	4,205	4,205	4,205	4,205	4,205
	Phrase 2	Correlation Coeff	−0.179^a^	1	−0.021	−0.236^a^	0.807^a^	0.016
		Sig. (bilateral)	0		0.183	0	0	0.308
		N	4,205	4,205	4,205	4,205	4,205	4,205
	Phrase 3	Correlation Coeff	0.056^a^	−0.021	1	0.098^a^	−0.059^a^	0.683^a^
		Sig. (bilateral)	0	0.183		0	0	0
		N	4,205	4,205	4,205	4,205	4,205	4,205
	Phrase 4	Correlation Coeff	0.761^a^	−0.236^a^	0.098^a^	1	−0.234^a^	0.095^a^
		Sig. (bilateral)	0	0	0		0	0
		N	4,205	4,205	4,205	4,205	4,205	4,205
	Phrase 5	Correlation Coeff	−0.190^a^	0.807^a^	−0.059^a^	−0.234^a^	1	0.035^b^
		Sig. (bilateral)	0	0	0	0		0.024
		N	4,205	4,205	4,205	4,205	4,205	4,205
	Phrase 6	Correlation Coeff	0.069^a^	0.016	0.683^a^	0.095^a^	0.035^b^	1
		Sig. (bilateral)	0	0.308	0	0	0.024	
		N	4,205	4,205	4,205	4,205	4,205	4,205

^a^ Correlation is significant at the level 0.01 (bilateral).

^b^ Correlation is significant at the level 0.05 (bilateral).

Source: The authors.

Most correlations between options are weak, which means that there is no particular reason why someone would prefer or prefer not to say “chiques” and “amigos y amigas”; in other words, the absence of correlation between these items can be interpreted as all of them being part of a repertoire that can be activated according to the situation, instead of being structurally co-dependent. The only two exceptions are 1 and 4, 2 and 5, and 3 and 6 (in bold in Table 4), which show strong correlations that make them directly proportional. In other words: someone’s attitude toward non-binary non-standard option “*chiques*” as a vocative will be the same if that form is being used in a non-vocative position. The same is true of generic masculine or binary standard forms: if a speaker is willing to use “chicos” or “chicos y chicas” as a vocative, they will have the same attitude in a non-vocative position; if a speaker finds its use unacceptable as a vocative, they will find it unacceptable in any other position.

This table also shows that there are low correlations among options that have the same position but differ in form, while there are high correlations between options that have the same form different positions. The fact that phrases with a different form in the same position are weakly or non-correlated seems to show the relative independence of these forms; that is, that accepting and/or adopting “chiques” is not a reason to reject or not use “chicos” or “chicas y chicos.. Therefore, we can understand this relative independence as a sign of being a part of a repertoire that does not privilege or prefer one option over the others. On the contrary, we can hypothesize that a future qualitative study would show that the effective adoption of one form or the other will be contextually dependent. Although it could be interpreted that this matrix globally argues against Hypothesis 2, as there exist strong correlations between the same form in both positions, crosstabs will show that the percentage of attitudes -especially with regard to non-binary non-standard forms-differ in about 10%. Therefore, although they are directly proportional, they show different frequencies.

In the following section, attitudes of the total sample toward the six phrases will be analyzed. For the sake of clarity, results were grouped according to acceptability and adoptability.

### Attitudes Toward Non-binary Non-standard Options: *Chiques*/*Amigues*

The general data (i.e., without distinguishing by gender, place of residence, age, etc.) indicate the following values of acceptance for the non-binary non-standard option, i.e., for “chiques”.

**FIGURE 1:**
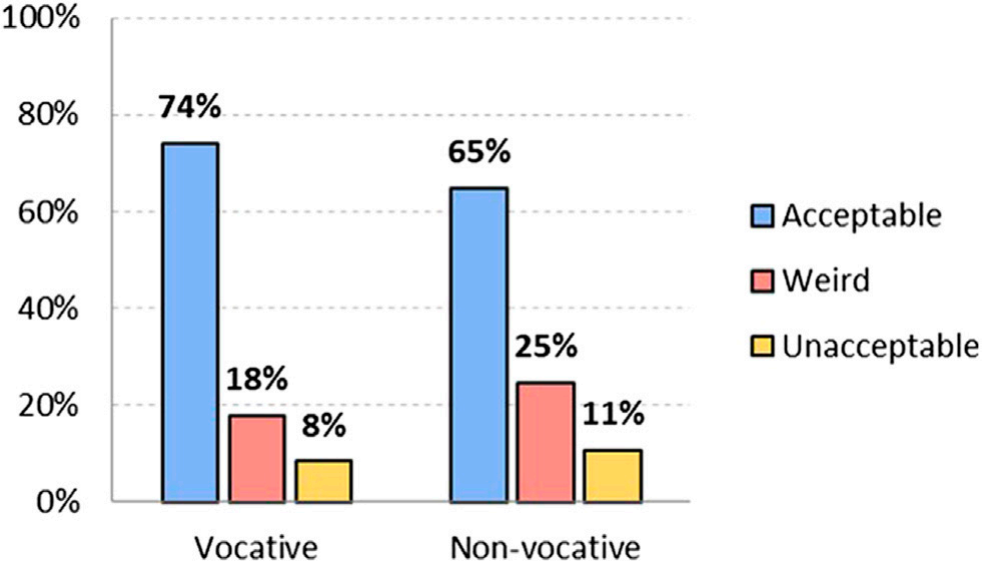
Level of acceptability of non-binary non-standard *option chiques*, both vocative and non-vocative use (in %). Source: The authors.

As seen in Figure 1, in the vocative position, at the beginning of the sentence, 74.2% of the respondents find the non-binary, non-standard option acceptable, 17.6% find it “weird” and 8.3% consider it unacceptable. This indicates that a large number of the survey respondents accept the use of inclusive language in different positions. Hypothesis 2 proposed that “chiques” is more acceptable in the vocative position than in the middle of the sentence, and this difference is verified by 10 points: 74.2% in the vocative position, and 64.9% in the non-vocative position. There are more people who find its use in the non-vocative position weird (24.6%) or unacceptable (10.5%).

Gender identification behaves as expected. Non-binary respondents find it acceptable in vocative position by 84%, compared to 68.5% of men and 76.3% of women. It is found weird by 11.5% of non-binary, 20.9% of men and 16.4% of women. Finally, it is unacceptable to 4.3% of non-binary, 10.6% of men and 7.3% of women.

In a non-vocative position, non-binary respondents still show higher acceptance than the average: 84% find it acceptable and 10.1% find it weird. Men, on the other hand, find it unacceptable in 15%, weird in 28.8% and acceptable in 56.2%. Finally, women accept the non-binary non-standard option in non-vocative position in 67.9%, find it weird in 23.2% and unacceptable in 8.8%. Thus, in general terms, gender is significant: in the case of non-binary, positive attitudes (of acceptance and willingness to use) are much higher than average, while men’s negative attitudes (of non-acceptance and non-use) are higher. Women are usually slightly more positive and less negative than the total sample.

**FIGURE 2:**
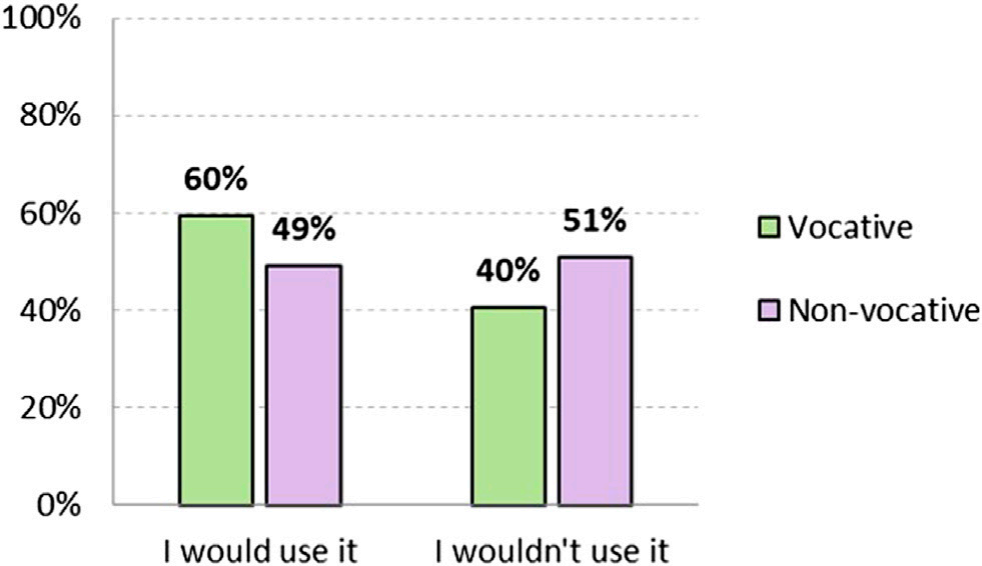
Level of adoptability of non-binary non-standard option *chiques*, both vocative and non-vocative use (in %). Source: The authors.

Hypothesis 1 proposed that accepting the use of *chiques* in other people is not the same as being willing to use it oneself. Thus as shown in Figure 2, although 59.6% would use it in a vocative position, only 49.1% would do so in a non-vocative position. In both cases, there is a difference of almost 15 points less with respect to acceptability. These results show that there is an attitude that could be described as "tolerant" toward non-binary language: speakers accept its use by others, but are not willing to use it themselves. Acceptability is higher in vocative position, and remarkably lower in non-vocative position, where rejection (non-acceptance) increases from 8.3% to 10.5%.

When analyzed according to gender, non-binary respondents would use it in a vocative position in 87.1%, followed by 64.6% of women, and 48.2% of men. In non-vocative positions, attitudes are less positive, as expected: 72.4% of non-binary, 52.9% of women and 38.5% of men. As in the case of acceptability, differences according to gender identification are significant.

### Attitudes Toward Generic Masculine: *Chicos*/*Amigos*

**FIGURE 3:**
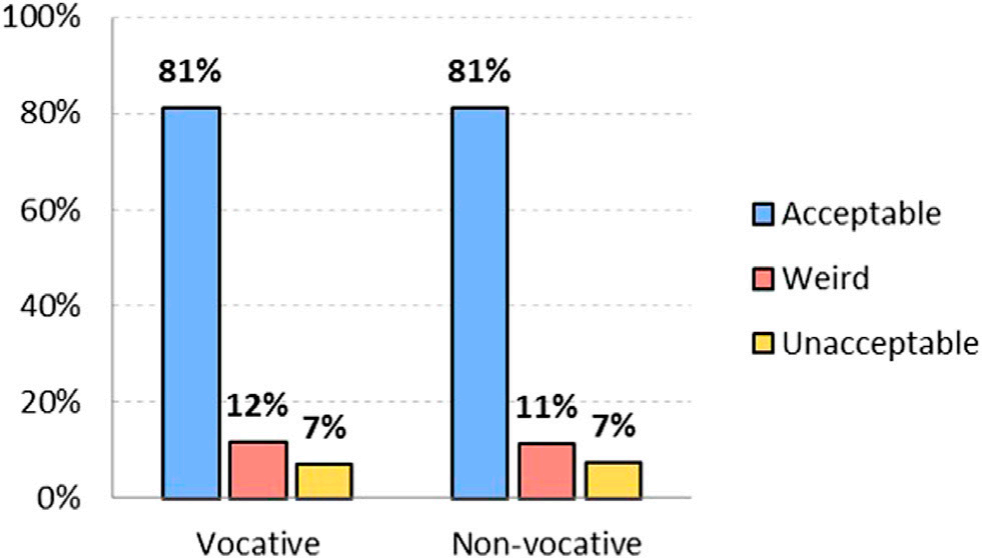
Level of acceptability of generic masculine option *chicos/amigos*, both vocative and non-vocative use (in %). Source: The authors.

One of the fears that the use of NGL arouses among its detractors is that it will “deform” the language, i.e., that those who use it will abandon the standard morphology of grammatical gender. Results show that that fear is unjustified, because attitudes toward the use of the generic masculine show high levels of acceptability:

Figure 3 shows that the option of the generic masculine is the most widely accepted, both in the vocative position (81.2% find it acceptable, against 11.6% to whom it sounds weird and 7.2% who consider it unacceptable) and in a non-vocative position (which 81.4% find acceptable, 11.4% find weird and 7.3% find unacceptable). In this case, the attitude toward generic masculine does not change whether or not it is used in a vocative position.

Attitudes of non-binary respondents show that it is not perceived as unacceptable, although they would not use it. In the vocative position, only 11.5% of non-binary respondents find it unacceptable (and 20.3% find it weird), compared to 4.1% (and 7.6%) of men and 8.3% (and 13.2%) of women. In a non-vocative position, the generic masculine “*amigos”* is found to be unacceptable only by 11.5% of non-binary respondents (weird by 24.6% and acceptable by 63.8%), followed by women (78.5% acceptable, 13% weird, and 8.5% unacceptable) and men (89.1% acceptable, 6.8% weird, and 4.2% unacceptable). In the case of generic masculine, as expected, non-binary and women have more-than-the-average negative attitudes than men, much higher in the case of non-binary.

**FIGURE 4:**
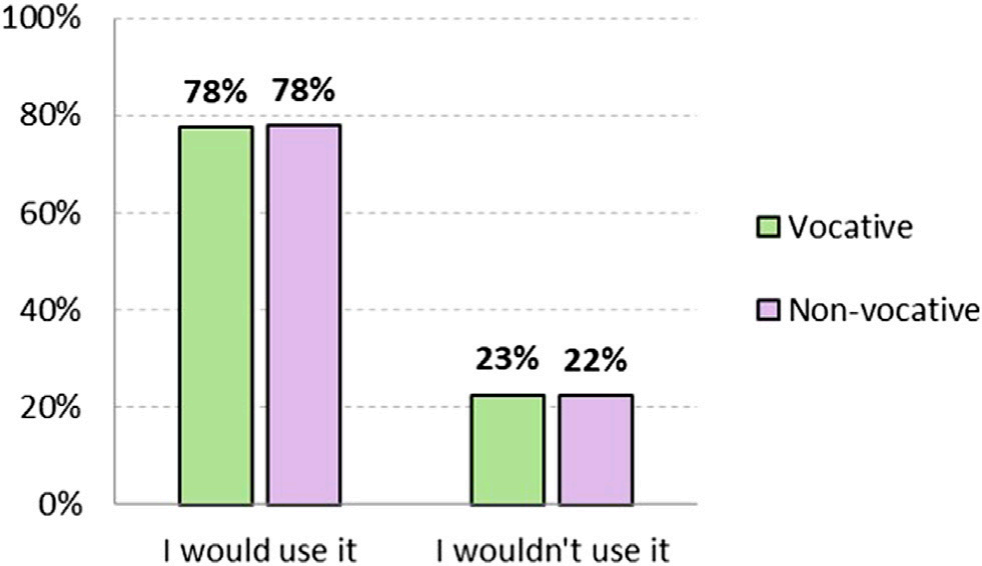
Level of adoptability of generic masculine option *chicos/amigos*, both vocative and non-vocative use (in %). Source: The authors.

To a slightly lesser extent, but with little significant difference, generic masculine is also the form that would be used most, as seen in Figure 4: 77.5% as vocative and 77.9% as non-vocative. Those who would not use it also maintain a similar attitude in both positions: they have 22.5% rejection as vocative and 22.2% in the non-vocative position.

From the perspective of gender, 54.9% of non-binary respondents would use generic masculine in a vocative position, followed by 75.8% of women and 83.3% of men. In a non-vocative position, the situation is similar: 52.1% of non-binary would use it, followed by women (75.5%) and men (84.9%). Again, gender is closely related to attitudes toward generic masculine, as shown by the more positive attitude of binary than non-binary gender identification, and of men than women.

It is worth noting that, unlike the non-binary option, in the case of the generic masculine there is no difference in attitude according to its position (vocative or non-vocative); i.e., it has the same level of acceptance or rejection, and of willingness or unwillingness to use it, in both positions.

This is an expected result: since it is the unmarked option, i.e., the one that is acquired when the language is learned, it sounds equally good in any position. The non-binary option, on the other hand, sounds better where it is used more strategically: at the beginning, as a vocative.

### Attitudes Toward Binary Standard Forms: *Chicos y Chicas*/*Amigos y Amigas*

What happened to the more inclusive, but still binary, standard option: “boys and girls,” “*chicos y chicas*”?

**FIGURE 5:**
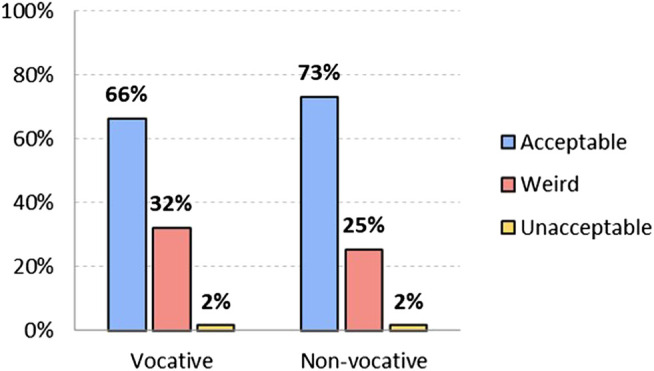
Level of acceptability of binary standard option *chicos y chicas/amigos y amigas*, both vocative and non-vocative use (in %). Source: The authors.

This binary standard option is not as conservative as the generic masculine, but neither is it innovative in linguistic or gender terms. Unlike the previous options, it sounds quite weird: although 66.3% find it acceptable as vocative, 32.1% find it weird and almost no-one (1.6%) finds it unacceptable. In the non-vocative position, on the other hand, it is more widely accepted (73.2%), less weird (25.3%) and equally unacceptable (1.6%) (see Figure 5). These results clearly show that it is more acceptable in the middle of the sentence than at the beginning.

From the perspective of gender identification, the binary option in vocative position is seen as acceptable especially by women (67.8%), followed by men (63.6%) and non-binary (in a surprisingly high 50.7%, although still very much lower than the average). The relationship is reversed in the case of weirdness: 44.9% of non-binary, 34.8% of men, and 30.7% of women. Finally, as expected, it is seen as unacceptable mostly by non-binary respondents (4.3%), although it was expected a higher rejection rate of the binary from a non-binary perspective. Attitudes regarding this option in a non-vocative position are very similar to those for the vocative position, showing approximately the same percentages as for vocative position.

**FIGURE 6:**
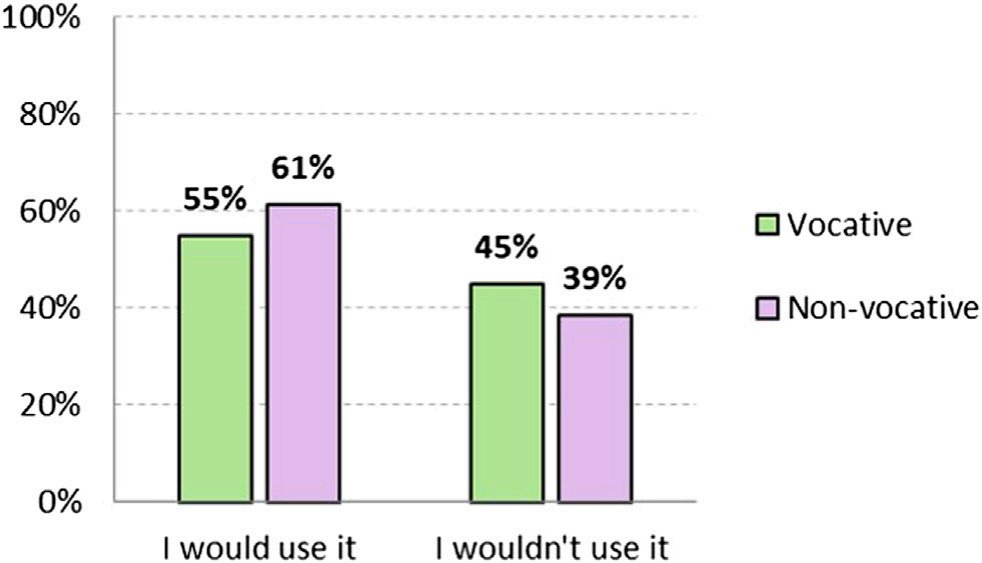
Level of adoptability of binary standard option *chicos y chicas/amigos y amigas*, both vocative and non-vocative use (in %). Source: The authors.

In terms of use, Figure 6 shows that, in there are also more people who are willing to use the binary standard form in a non-vocative position (61.4%) than in a vocative one (55%). However, the number of people who would not use it is very high: 45% in a vocative position and 38.7% in a non-vocative position.

From the perspective of gender identification, the binary standard option is more adoptable in a vocative position by women (56.7%), followed by men (51.7%), and non-binary (37.6%). In a non-vocative position, it is more adoptable by women (63.7%), followed by men (57.1%) and non-binary (39.1%). In this case, women have a more than average positive attitude toward its use, either in a vocative or non-vocative position. As in the previous analyses, there are relatively high levels of adoptability of the binary standard form in non-binary speakers.

Similar to the non-binary non-standard option, “chiques,” the binary standard option generates more acceptability than adoptability. The reason, however, is probably the reverse: some people accept it because they know it is “correct”, but it sounds so bad that they do not want to say it.

## Discussion

As pointed out earlier, the main limitation of this study is its very restricted survey design, where only six phrases were evaluated, due to the need for a short form to avoid the usually high levels of attrition of web-based surveys. A second limitation is regarding to sampling: as it is a non-probabilistic convenience sample, thus, results cannot be generalized to the population of Argentina. In the same way, other sampling biases prevent us from studying whether the event is typical of the most cosmopolitan cities or not. However, significant differences and association between analyzed variables are sufficient to be theoretically relevant.

The two hypotheses tested in this study seemed to be proven by the results.

As stated, difference between acceptability and adoptability was observed in the evaluation of the three forms (non-binary, masculine and binary) in both two positions (vocative and non-vocative). The innovative (non-binary) form was, to speakers in general, more acceptable than adoptable. The opposite is true for the generic masculine: although it is slightly less acceptable, it is largely adoptable. This is a consequence of and evidence that the generic masculine still works as the non-marked grammatical gender in Spanish.

The non-vocative position was the one that least accepts innovation; on the contrary, the place at the beginning of the sentence is where the non-binary option, "chiques", was more accepted. The vocative can be interpreted as helping to propose an identity and define the speaker and the addressee, as a typical phenomenon of social deixis. Saying "chiques" takes more cognitive effort, because it is the marked, non-standard option. However, using it creates an interpersonal relationship, where the speakers recognize each other as people who share a non-binary conception of gender, even when they are not willing to use it extensively in their speech.

Is this a process of grammatization of a third gender in Spanish? This is a question that few scholars ask, and even fewer can answer. The fact that it is far more accepted as a vocative, in a peripheral part of the phrase, seems to show that it is not. Furthermore, the high levels of acceptability and adoptability of generic masculine, not only by the general population, but also when seen from the perspective of gender identification, show that the form is very much alive. However, the significant differences observed according to gender show that the marks of grammatical gender are closely related to gender identification. In this case, attitudes were as expected, with a more conservative tendency in the case of men, and a more disruptive one in the case of non-binary respondents. Women, on the other hand, behave slightly more like non-binary people in the cases of generic masculine and non-standard non binary *chiques*. However, women’s attitudes differ in the case of binary standard options (“*chicos y chicas*” and “*amigos y amigas*”), which seemed to be more acceptable and adoptable to women than to the rest of the sample.

Are inclusive language activists a group of purists that want to impose such language on everybody? The high levels of acceptability and adoptability of all forms show that they are not mutually exclusive, but a part of a repertoire. In a future study I will analyze this aspect in greater depth by building a typology of speakers according to their attitudes toward the three forms proposed.

Finally, we think our study allows for a more nuanced approach to the study of linguistic attitudes. By avoiding the “positive/neutral/negative” scale we can understand attitudes not only in terms of value judgments, but in terms of acceptance. Thus, attitudes are not only opinions toward linguistic forms, but attitudes toward speakers, as people often can accept in others something they do not like personally or are not willing to adopt. In second place, by evaluating vocative/non-vocative positions we have been able to understand the pragmatic impact of syntax in linguistic attitudes. This is especially important as many research in the field address linguistic forms de-contextualized from verbal context. Finally, with regard to research on GNL and ideological motivations, we observe the role of linguistic ideologies in shaping attitudes toward non-standard non-binary forms. The distance between accepting other speakers’ use of non-standard non-binary *chiques* but resisting its adoption in one’s own speech shows a non binary political (i.e. gender) ideology, but a more conservative linguistic ideology. Further investigation is required, in this aspect, to understand the social motivations, and their impacts, behind this attitude toward non-standard non-binary forms.

Unlike other surveys on the matter (e.g. Gustaffson Sendén et al., 2015 ), we did not ask for “use”, as it is often not transparent to speakers and is more difficult to account for. Kalinowski (2020a) shows that only 33% of Argentine Twitter users employed a non-binary non-standard form during 2019, without distinguishing legitimate uses from quotations, parodies, etc. The data in the current study, on the contrary, show that 60% of respondents would use it, at least in the vocative position. This means that reported use does not reflect actual use, and that a question thus formulated (“Do you use it?”) can be ambiguous. Thus, defining it as willingness to use, or adoptability, makes it clearer that the respondent is assessing willingness to act, not the action in itself, and this could be a methodological asset for future research on linguistic attitudes.

We find necessary, for future research, to explore contextual factors conditioning the adoption of non-binary non-standard forms through qualitative sociolinguistic studies. This will allow for understanding how these forms are selected and used in actual settings. Furthermore, in-depth interviews would allow for better understand what “acceptance/weirdess/non-acceptance” means to speakers, especially in those who are not willing to use these forms. Finally, cross-cultural comparison will help deepening the social motivations of these attitudes, especially with regard to place of residence (rural/urban) and nationality.

## Data Availability

The raw data supporting the conclusion of this article will be made available by the authors, without undue reservation.

## Footnotes

1 Due to this modality of participation, it is not possible to record the number of actual invitations. Moreover, the technique of data entry (Google Forms) does not allow to know how many participants abandoned the questionnaire before completion. Therefore, there are not invalid or dropped cases in our sample.

2 Crosstabs were tested using the non-parametric chi-square test. A significance level of 95% was chosen for the entire data analysis procedure.

3 As shown, non-binary group clearly surpasses the minimum of *n* = 30 required for considering large samples procedures (for instance, the Central Limit Theorem) at the same time that having n = 69 (crearly a very small sample proportion compared to other genders) did not threatened in any case the statistical assumption needed for the validity of chi-square test, that is, that no more than 25% of cells should have an expected value below 5.

4 As applied in the rest of this article, when crosstabs were used, data was chi-square tested at a 95% significance level.

## References

[B1] Ansara Y. G. Hegarty P. (2014). Methodologies of misgendering: recommendations for reducing cisgenderism in psychological research. Fem. Psychol. 24, 259–270. 10.1177/0959353514526217

[B2] Barrera Linares L. (2019). Relación género/sexo y masculino inclusivo plural. Lit. Lingüíst. 40, 327–354. 10.29344/0717621x.40.2070

[B3] Beatty Martínez A. L. Dussias P. E. (2019). Revisiting masculine and feminine grammatical gender in Spanish: linguistic, psycholinguistic, and neurolinguistic evidence. Front. Front. Psychol. 10, 751. 10.3389/fpsyg.2019.00751 31024394 PMC6460095

[B4] Berger M. (2019). A guide to how gender-neutral language is developing in the world. the guardian. Available at: https://www.washingtonpost.com/world/2019/12/15/guide-how-gender-neutral-language-is-developing-around-world/ (Accessed November 15, 2020).

[B5] Binkey C. (2015). He? She? Ze? Colleges add gender-free pronouns, alter policy. Available at: https://apnews.com/article/48c986c722ba4e5bb8a5a4c1f1d31df1 (Accessed November 15, 2020).

[B6] Bolívar A. (2019). Una introducción al análisis crítico del lenguaje inclusivo. Lit. Lingüíst. 40, 355–375. 10.29344/0717621X.40.2071

[B7] Boroditsky L. Schmidt L. A. Phillips W. (2003). “Sex, syntax, and semantics,” in Language in mind: advances in the study of language and thought. Editors Getner D. Goldin-Meadow S. (Cambridge, MA: MIT Press), 61–79.

[B8] Brutt-Griffler J. Kim S. (2017). In their own voices: development of English as a gender-neutral language. English Today. 34 (1), 12–19. 10.1017/S0266078417000372

[B9] Castillo Sánchez S. Mayo S. (2019). El lenguaje inclusivo como “norma” de empatía e identidad: reflexiones entre docentes y futures profesores. Lit. Lingüíst. 40, 377–391. 10.29344/0717621X.40.2072

[B10] Chávez Fajardo S. (2019). Ginopia, silencio. Género, discurso, diccionario. Lit. Lingüíst. 40, 393–429. 10.29344/0717621X.40.2073

[B11] Darr B. Kibbey T. (2016). Pronouns and thoughts on neutrality: gender concerns in modern grammar. Pursuit: J. Undergraduate Res. Univ. Tenn. 7 (1), 71–84.

[B12] Everett C. (2011). Gender, pronouns and thought: the ligature between epicene pronouns and a more neutral gender perception. G&L. 5 (1), 133–152. 10.1558/genl.v5i1.133

[B13] Fletcher L. (1988). El sexismo lingüístico y su uso acerca de la mujer. Feminaria. 1 (1), 29–33.

[B14] Gasparri J. (2020). “Acerca del lenguaje inclusivo: cuestiones teóricas, razones políticas,” in Apuntes sobre lenguaje no sexista e inclusivo. Editors Kalinowski S. Gasparri J. Pérez S. I. Moragas F. (Rosario: UNR Editora), 31–67.

[B15] Glozman M. (2020). Lenguaje y movimiento feminista: crítica del idealismo lingüístico. Revista Zigurat. Available at: https://revistazigurat.com.ar/lenguaje-y-movimiento-feminista-critica-del-idealismo-linguistico/ (Accessed November 15, 2020).

[B16] Gustaffson Sendén M. Bäck E. A. Lindqvist A. (2015). Introducing a gender-neutral pronoun in a natural gender language: the influence of time on attitudes and behavior. Front. Psychol. 6, 893. 10.3389/fpsyg.2015.00893 26191016 PMC4486751

[B17] Hochheimer C. J. Sabo R. T. Krist A. H. Day T. Cyrus J. Woolf S. H. (2016). Methods for evaluating respondent attrition in web-based surveys. J. Med. Internet Res. 18 (11), e301. 10.2196/jmir.6342 27876687 PMC5141338

[B18] Imborek K. L. Nisly N. L. Hesseltine M. J. Grienke J. Zikmund T. A. Dreyer N. R. (2017). Preferred names, preferred pronouns, and gender identity in the electronic medical record and laboratory information system: is pathology ready? J. Pathol. Inform. 8 (8), 42. 10.4103/jpi.jpi_52_17 29114436 PMC5653959

[B19] Kalinowski S. (2020a). Lenguaje inclusivo en usuarios de Twitter en Argentina: un estudio de corpus. Cuarenta Naipes. 2 (3), 233–259.

[B20] Kalinowski S. (2019). Lenguaje inclusivo: cambio lingüístico o cambio social. Revista CTPCBA. 141, 53–55.

[B21] Kalinowski S. (2020b). “Lenguaje inclusivo: configuración discursiva de varias luchas,” in Apuntes sobre lenguaje no sexista e inclusivo. Editors Kalinowski S. Gasparri J. Pérez S. I. Moragas F. (Rosario: UNR Editora), 17–29.

[B22] Martínez A. (2019). Disidencias en la conformación de la gramática: el lenguaje inclusivo. Revista Heterotopías. 2 (4), 1–16.

[B23] Parks R. Straka R. (2018). “Gender pronouns to support identity: creating a campus of difference,” in Phi Kappa Phi Forum, Fall 2018, Vol. 98, 8–11.

[B24] Pérez S. Moragas F. (2020). “Lenguaje inclusivo: malestares y resistencias en el discurso conservador,” in Apuntes sobre lenguaje no sexista e inclusivo. Editors Kalinowski S. Gasparri J. Pérez S. I. Moragas F. (Rosario: UNR Editora), 69–95.

[B25] Popic D. Gorjanc V. (2018). Challenges of adopting gender-inclusive language in Slovene. SL. 86, 329–350. 10.22210/suvlin.2018.086.07

[B33] Real Academia Española (2010). Nueva gramática de la lengua española. Manual. Madrid, Spain, United States:RAE.

[B26] Romero M. C. Funes M. S. (2018). Nuevas conceptualizaciones de género en el español de la Argentina: un análisis cognitivo-prototípico. RASAL-Lingüística. 2018, 7–39.

[B27] Sayago S. (2019). Apuntes sociolingüísticos sobre el lenguaje inclusivo. RevCom. 9, e015. 10.24215/24517836e0152019

[B28] Schmidt S. (2019). A language for all. The Wash. Post. Available at: https://www.washingtonpost.com/dc-md-va/2019/12/05/teens-argentina-are-leading-charge-gender-neutral-language/?arc404=true (Accessed November 15, 2020).

[B29] Shormani M. Q. Quarabesh A. (2018). Vocatives: correlating the syntax and discourse at the interface. Cogent Arts and Humanities. 5 (1), 1469388. 10.1080/23311983.2018.1469388

[B30] Stahlberg D. Braun F. Irmen L. Sczesny S. (2007). “Representation of the sexes in language,” in Social communication. Editor Fiedler K. (New York: Psychology Press), 163–187.

[B31] Tosi C. L. (2020). ¿Hojas de estilo para el lenguaje inclusivo? Un análisis acerca de las prácticas de corrección de estilo en el ámbito editorial. Exlibris. 9, 169–179.

[B32] Tosi C. L. (2019). Marcas discursivas de la diversidad. Alabe. 10, 1. 10.15645/Alabe2019.20.11

