# Supplementary material for: Attitudes Toward Gender-Neutral Spanish: Acceptability and Adoptability
Source: Front Sociol. 2021 Mar 15;6:629616. doi: 10.3389/fsoc.2021.629616 (PMC8022528; doi:10.3389/fsoc.2021.629616)
Supplement: Supplementary file 1 [file datasheet1.pdf]

ANNEX 1

Form 1 (Translated)

Attitudes towards gender marking in language

Hello! Thank you for participating in our survey. The objective is to know what people who speak Spanish in Argentina think about different ways of addressing a mixed group through WhatsApp audios.
Our interest is only scientific, and we are interested in documenting in the best possible way what people think about the way we use language.
All responses are very important and valuable to our work, and that is why we appreciate the greatest possible honesty.
The responses are anonymous.

For any question or suggestion, you can contact us by e-mail at encuestaslinguistica@gmail.com.

Thanks a lot!

Gender:

- Feminine
- Masculine
- Non-binary
- I prefer not to tell

Age:
(Drop down list: 12 – 99)

Place of residence:
(Drop down list)

- Buenos Aires
- Catamarca
- Chaco
- Chubut
- Ciudad Autónoma de Buenos Aires
- Córdoba
- Corrientes
- Entre Ríos
- Formosa
- Gran Buenos Aires - Norte
- Gran Buenos Aires – Oeste
- Gran Buenos Aires - Sur
- Jujuy
- La Pampa
- La Rioja
- Mendoza
- Misiones
- Neuquén
- Río Negro
- Salta

- San Juan
- Santa Cruz
- Santa Fe
- Santiago del Estero
- Tierra del Fuego
- Tucumán

Educational level:

- Primary incomplete
- Primary complete
- Secondary incomplete
- Secondary complete
- Tertiary/ University incomplete
- Tertiary/ University complete
- Postgraduate incomplete
- Postgraduate complete

What is your attitude if you get the following audios to a mixed WhatsApp group?

Audio 1 (female voice): “Hey, chiques, ¿quieren venir al cine esta noche?”

- I find it acceptable and I would use it.
- I find it acceptable but I would not use it.
- I find it weird but I would use it.
- I find it weird and I wouldn’t use it.
- I find it unacceptable and I wouldn’t use it.
- I find it unacceptable, but I would use it.

Audio 2 (female voice): “Hey, chicos, ¿quieren venir al cine esta noche?”

- I find it acceptable and I would use it.
- I find it acceptable but I would not use it.
- I find it weird but I would use it.
- I find it weird and I wouldn’t use it.
- I find it unacceptable and I wouldn’t use it.
- I find it unacceptable, but I would use it.

Audio 3 (female voice): “Hey, chicos y chicas, ¿quieren venir al cine esta noche?”

- I find it acceptable and I would use it.
- I find it acceptable but I would not use it.
- I find it weird but I would use it.
- I find it weird and I wouldn’t use it.
- I find it unacceptable and I wouldn’t use it.
- I find it unacceptable, but I would use it.

Audio 4 (male voice): “Les dije a todes mis amigues que vinieran al cine”

- I find it acceptable and I would use it.
- I find it acceptable but I would not use it.
- I find it weird but I would use it.
- I find it weird and I wouldn’t use it.
- I find it unacceptable and I wouldn’t use it.
- I find it unacceptable, but I would use it.

Audio 5 (male voice): “Les dije a todos mis amigos que vinieran al cine”

- I find it acceptable and I would use it.
- I find it acceptable but I would not use it.
- I find it weird but I would use it.
- I find it weird and I wouldn’t use it.
- I find it unacceptable and I wouldn’t use it.
- I find it unacceptable, but I would use it.

Audio 6 (male voice): “Les dije a todos mis amigos y mis amigas que vinieran al cine”

- I find it acceptable and I would use it.
- I find it acceptable but I would not use it.
- I find it weird but I would use it.
- I find it weird and I wouldn’t use it.
- I find it unacceptable and I wouldn’t use it.
- I find it unacceptable, but I would use it.

Form 2 (Translated)

Attitudes towards gender marking in language

Hello! Thank you for participating in our survey. The objective is to know what people who speak Spanish in Argentina think about different ways of addressing a mixed group through WhatsApp audios.
Our interest is only scientific, and we are interested in documenting in the best possible way what people think about the way we use language.
All responses are very important and valuable to our work, and that is why we appreciate the greatest possible honesty.
The responses are anonymous.

For any question or suggestion, you can contact us by e-mail at encuestaslinguistica@gmail.com.

Thanks a lot!

Gender:

- Feminine
- Masculine
- Non-binary
- I prefer not to tell

Age:
(Drop down list: 12 – 99)

Place of residence:
(Drop down list)

- Buenos Aires
- Catamarca
- Chaco
- Chubut
- Ciudad Autónoma de Buenos Aires
- Córdoba
- Corrientes
- Entre Ríos
- Formosa
- Gran Buenos Aires - Norte
- Gran Buenos Aires – Oeste
- Gran Buenos Aires - Sur
- Jujuy
- La Pampa
- La Rioja
- Mendoza
- Misiones
- Neuquén
- Río Negro
- Salta
- San Juan
- Santa Cruz
- Santa Fe
- Santiago del Estero
- Tierra del Fuego
- Tucumán

Educational level:

- Primary incomplete
- Primary complete
- Secondary incomplete
- Secondary complete
- Tertiary/ University incomplete
- Tertiary/ University complete
- Postgraduate incomplete
- Postgraduate complete

What is your attitude if you get the following audios to a mixed WhatsApp group?

Audio 1 (male voice): "Hey, chiques, ¿quieren venir al cine esta noche?"

- I find it acceptable and I would use it.
- I find it acceptable but I would not use it.
- I find it weird but I would use it.
- I find it weird and I wouldn’t use it.
- I find it unacceptable and I wouldn’t use it.
- I find it unacceptable, but I would use it.

Audio 2 (male voice): “Hey, chicos, ¿quieren venir al cine esta noche?”

- I find it acceptable and I would use it.
- I find it acceptable but I would not use it.
- I find it weird but I would use it.
- I find it weird and I wouldn’t use it.
- I find it unacceptable and I wouldn’t use it.
- I find it unacceptable, but I would use it.

Audio 3 (male voice): “Hey, chicos y chicas, ¿quieren venir al cine esta noche?”

- I find it acceptable and I would use it.
- I find it acceptable but I would not use it.
- I find it weird but I would use it.
- I find it weird and I wouldn’t use it.
- I find it unacceptable and I wouldn’t use it.
- I find it unacceptable, but I would use it.

Audio 4 (female voice): “Les dije a todes mis amigues que vinieran al cine”

- I find it acceptable and I would use it.
- I find it acceptable but I would not use it.
- I find it weird but I would use it.
- I find it weird and I wouldn’t use it.
- I find it unacceptable and I wouldn’t use it.
- I find it unacceptable, but I would use it.

Audio 5 (female voice): “Les dije a todos mis amigos que vinieran al cine”

- I find it acceptable and I would use it.
- I find it acceptable but I would not use it.
- I find it weird but I would use it.
- I find it weird and I wouldn’t use it.
- I find it unacceptable and I wouldn’t use it.

- I find it unacceptable, but I would use it.

Audio 6 (female voice): “Les dije a todos mis amigos y mis amigas que vinieran al cine”

- I find it acceptable and I would use it.
- I find it acceptable but I would not use it.
- I find it weird but I would use it.
- I find it weird and I wouldn’t use it.
- I find it unacceptable and I wouldn’t use it.
- I find it unacceptable, but I would use it.
