# Supplementary material for: Attitudes Toward Gender-Neutral Spanish: Acceptability and Adoptability
Source: Front Sociol. 2021 Mar 15;6:629616. doi: 10.3389/fsoc.2021.629616 (PMC8022528; doi:10.3389/fsoc.2021.629616)
Supplement: Supplementary file 2 [file datasheet2.pdf]

ANNEX 2

(Part A)

TABLE 1. Acceptability and Adoptability of **<u>Attitude 1</u>** according to Form and Gender (absolute frequency and %).

b

			FORM 1*				FORM 2**			
			Feminine	Masculine	Non-binary	Total	Feminine	Masculine	Non-binary	Total
ATTITUDE 1	I find it unacceptable and I wouldn't use it	Count	87	54	0	141	94	72	3	169
		%	6,4	10,1	0	7,3	6,2	10,7	7,9	7,6
	I find it unacceptable, but I would use it	Count	8	1	0	9	22	2	0	24
		%	0,6	0,2	0	0,5	1,5	0,3	0	1,1
	I find it weird and I wouldn't use it	Count	128	69	2	199	157	107	3	267
		%	9,4	12,9	6,5	10,3	10,4	15,9	7,9	12,0
	I find it weird but I would use it	Count	96	31	1	128	92	45	2	139
		%	7,0	5,8	3,2	6,6	6,1	6,7	5,3	6,2
	I find it acceptable but I wouldn't use it	Count	269	138	2	409	311	179	7	497
		%	19,7	25,8	6,5	21,1	20,5	26,6	18,4	22,3
	I find it acceptable and I would use it	Count	780	242	26	1048	840	267	23	1130
		%	57,0	45,2	83,9	54,2	55,4	39,7	60,5	50,8
TOTAL		Count	1368	535	31	1934	1516	672	38	2226
		%	100,0	100,0	100,0	100,0	100,0	100,0	100,0	100,0

* p-value FORM 1:<0.000

** p-value FORM 2: <0.000

Source: The authors.

TABLE 2. Acceptability and Adoptability of **Attitude 2** according to FORM and Gender (absolute frequency and %).

			FORM 1*				FORM 2**			
			Feminine	Masculine	Non-binary	Total	Feminine	Masculine	Non-binary	Total
ATTITUDE 2	I find it unacceptable and I wouldn't use it	Count	44	9	2	55	58	8	3	69
		%	3,2	1,7	6,5	2,8	3,8	1,2	7,9	3,1
	I find it unacceptable, but I would use it	Count	64	15	2	81	75	17	1	93
		%	4,7	2,8	6,5	4,2	4,9	2,5	2,6	4,2
	I find it weird and I wouldn't use it	Count	83	13	4	100	80	17	4	101
		%	6,1	2,4	12,9	5,2	5,3	2,5	10,5	4,5
	I find it weird but I would use it	Count	103	25	3	131	112	36	3	151
		%	7,5	4,7	9,7	6,8	7,4	5,4	7,9	6,8
	I find it acceptable but I wouldn't use it	Count	213	67	8	288	222	89	12	323
		%	15,6	12,5	25,8	14,9	14,6	13,2	31,6	14,5
	I find it acceptable and I would use it	Count	861	406	12	1279	969	505	15	1489
		%	62,9	75,9	38,7	66,1	63,9	75,1	39,5	66,9
TOTAL		Count	1368	535	31	1934	1516	672	38	2226
		%	100,0	100,0	100,0	100,0	100,0	100,0	100,0	100,0

* p-value FORM 1:<0.000

** p-value FORM 2:<0.000

Source: The authors.

TABLE 3. Acceptability and Adoptability of **Attitude 3** according to FORM and Gender (absolute frequency and %).

			FORM 1*				FORM 2**			
			Feminine	Masculine	Non-binary	Total	Feminine	Masculine	Non-binary	Total
ATTITUDE 3	I find it unacceptable and I wouldn't use it	Count	14	5	2	21	17	10	1	28
		%	1,0	0,9	6,5	1,1	1,1	1,5	2,6	1,3
	I find it unacceptable, but I would use it	Count	6	2	0	8	6	2	0	8
		%	0,4	0,4	0	0,4	0,4	0,3	0	0,4
	I find it weird and I wouldn't use it	Count	255	142	12	409	275	140	8	423
		%	18,6	26,5	38,7	21,1	18,1	20,8	21,1	19,0
	I find it weird but I would use it	Count	182	60	4	246	174	78	7	259
		%	13,3	11,2	12,9	12,7	11,5	11,6	18,4	11,6
	I find it acceptable but I wouldn't use it	Count	330	121	6	457	356	164	14	534
		%	24,1	22,6	19,4	23,6	23,5	24,4	36,8	24,0
	I find it acceptable and I would use it	Count	581	205	7	793	688	278	8	974
		%	42,5	38,3	22,6	41,0	45,4	41,4	21,1	43,8
TOTAL		Count	1368	535	31	1934	1516	672	38	2226
		%	100,0	100,0	100,0	100,0	100,0	100,0	100,0	100,0

* p-value FORM 1: 0.001

** p-value FORM 2: 0.181

Source: The authors.

TABLE 4. Acceptability and Adoptability of **Attitude 4** according to FORM and Gender (absolute frequency and %).

			FORM 1*				FORM 2**			
			Feminine	Masculine	Non-binary	Total	Feminine	Masculine	Non-binary	Total
ATTITUDE 4	I find it unacceptable and I wouldn't use it	Count	117	76	1	194	110	93	3	206
		%	8,6	14,2	3,2	10,0	7,3	13,8	7,9	9,3
	I find it unacceptable, but I would use it	Count	15	4	0	19	11	8	0	19
		%	1,1	0,7	0	1,0	0,7	1,2	0	0,9
	I find it weird and I wouldn't use it	Count	193	120	2	315	212	127	4	343
		%	14,1	22,4	6,5	16,3	14,0	18,9	10,5	15,4
	I find it weird but I would use it	Count	150	47	1	198	116	53	0	169
		%	11,0	8,8	3,2	10,2	7,7	7,9	0	7,6
	I find it acceptable but I wouldn't use it	Count	330	139	4	473	395	187	5	587
		%	24,1	26,0	12,9	24,5	26,1	27,8	13,2	26,4
	I find it acceptable and I would use it	Count	563	149	23	735	672	204	26	902
		%	41,2	27,9	74,2	38,0	44,3	30,4	68,4	40,5
TOTAL		Count	1368	535	31	1934	1516	672	38	2226
		%	100,0	100,0	100,0	100,0	100,0	100,0	100,0	100,0

* p-value FORM 1:<0.000

** p-value FORM 2:<0.000

Source: The authors.

TABLE 5. Acceptability and Adoptability of **Attitude 5** according to FORM and Gender (absolute frequency and %).

			FORM 1*				FORM 2**			
			Feminine	Masculine	Non-binary	Total	Feminine	Masculine	Non-binary	Total
ATTITUDE 5	I find it unacceptable and I wouldn't use it	Count	59	10	3	72	60	8	2	70
		%	4,3	1,9	9,7	3,7	4,0	1,2	5,3	3,1
	I find it unacceptable, but I would use it	Count	64	18	1	83	63	14	2	79
		%	4,7	3,4	3,2	4,3	4,2	2,1	5,3	3,5
	I find it weird and I wouldn't use it	Count	78	12	5	95	78	14	5	97
		%	5,7	2,2	16,1	4,9	5,1	2,1	13,2	4,4
	I find it weird but I would use it	Count	105	23	4	132	114	32	3	149
		%	7,7	4,3	12,9	6,8	7,5	4,8	7,9	6,7
	I find it acceptable but I wouldn't use it	Count	211	62	8	281	221	77	10	308
		%	15,4	11,6	25,8	14,5	14,6	11,5	26,3	13,8
	I find it acceptable and I would use it	Count	851	410	10	1271	980	527	16	1523
		%	62,2	76,6	32,3	65,7	64,6	78,4	42,1	68,4
TOTAL		Count	1368	535	31	1934	1516	672	38	2226
		%	100,0	100,0	100,0	100,0	100,0	100,0	100,0	100,0

* p-value FORM 1:<0.000

** p-value FORM 2:<0.000

Source: The authors.

TABLE 6. Acceptability and Adoptability of **Attitude 6** according to FORM and Gender (absolute frequency and %).

			FORM 1*				FORM 2**			
			Feminine	Masculine	Non-binary	Total	Feminine	Masculine	Non-binary	Total
ATTITUDE 6	I find it unacceptable and I wouldn't use it	Count	11	7	1	19	20	8	1	29
		%	0,8	1,3	3,2	1	1,3	1,2	2,6	1,3
	I find it unacceptable, but I would use it	Count	5	3	1	9	6	1	0	7
		%	0,4	0,6	3,2	0,5	0,4	0,1	0	0,3
	I find it weird and I wouldn't use it	Count	214	105	10	329	175	104	7	286
		%	15,6	19,6	32,3	17	11,5	15,5	18,4	12,8
	I find it weird but I would use it	Count	174	52	5	231	148	56	3	207
		%	12,7	9,7	16,1	11,9	9,8	8,3	7,9	9,3
	I find it acceptable but I wouldn't use it	Count	285	128	7	420	343	165	16	524
		%	20,8	23,9	22,6	21,7	22,6	24,6	42,1	23,5
	I find it acceptable and I would use it	Count	679	240	7	926	824	338	11	1173
		%	49,6	44,9	22,6	47,9	54,4	50,3	28,9	52,7
TOTAL		Count	1368	535	31	1934	1516	672	38	2226
		%	100	100	100	100	100	100	100	100

* p-value FORM 1: 0.003

** p-value FORM 2: 0.019

Source: The authors.

(Part B)

TABLE 1. Acceptability and Adoptability of **Attitude 1** according to Gender* (in %).

			GENDER			TOTAL
			Feminine	Masculine	Non-binary	
ATTITUDE 1	I find it unacceptable and I wouldn't use it	Count	181	126	3	310
		%	6,3	10,4	4,3	7,5
	I find it unacceptable, but I would use it	Count	30	3	0	33
		%	1,0	0,2	0	0,8
	I find it weird and I wouldn't use it	Count	285	176	5	466
		%	9,9	14,6	7,2	11,2
	I find it weird but I would use it	Count	188	76	3	267
		%	6,5	6,3	4,3	6,4
	I find it acceptable but I	Count	580	317	9	906
		%	20,1	26,3	13,0	21,8

	I find it acceptable and I would use it	Count	1620	509	49	2178
		%	56,2	42,2	71,0	52,4
TOTAL		Count	2884	1207	69	4160
		%	100,0	100,0	100,0	100,0

* p-value: <0.000

Source: The authors.

TABLE 2. Acceptability and Adoptability of **Attitude 2** according to Gender* (in %).

			GENDER			TOTAL
			Feminine	Masculine	Non-binary	
ATTITUDE 2	I find it unacceptable and I wouldn't use it	Count	102	17	5	124
		%	3,5	1,4	7,2	3,0
	I find it unacceptable, but I would use it	Count	139	32	3	174
		%	4,8	2,7	4,3	4,2
	I find it weird and I wouldn't use it	Count	163	30	8	201
		%	5,7	2,5	11,6	4,8
	I find it weird but I would use it	Count	215	61	6	282
		%	7,5	5,1	8,7	6,8
	I find it acceptable but I wouldn't use	Count	435	156	20	611
		%	15,1	12,9	29,0	14,7

	it					
	I find it acceptable and I would use it	Count	1830	911	27	2768
		%	63,5	75,5	39,1	66,5
TOTAL		Count	2884	1207	69	4160
		%	100,0	100,0	100,0	100,0

* p-value: <0.000

Source: The authors.

TABLE 3. Acceptability and Adoptability of **Attitude 3** according to Gender* (in %).

			GENDER			TOTAL
			Feminine	Masculine	Non-binary	
ATTITUDE 3	I find it unacceptable and I wouldn't use it	Count	31	15	3	49
		%	1,1	1,2	4,3	1,2
	I find it unacceptable, but I would use it	Count	12	4	0	16
		%	0,4	0,3	0	0,4
	I find it weird and I wouldn't use it	Count	530	282	20	832
		%	18,4	23,4	29,0	20,0
	I find it weird but I would use it	Count	356	138	11	505
		%	12,3	11,4	15,9	12,1
	I find it acceptable but I wouldn't use it	Count	686	285	20	991
		%	23,8	23,6	29,0	23,8

	I find it acceptable and I would use it	Count	1269	483	15	1767
		%	44,0	40,0	21,7	42,5
TOTAL		Count	2884	1207	69	4160
		%	100,0	100,0	100,0	100,0

* p-value: <0.000

Source: The authors.

TABLE 4. Acceptability and Adoptability of **Attitude 4** according to Gender* (in %).

			GENDER			TOTAL
			Feminine	Masculine	Non-binary	
ATTITUDE 4	I find it unacceptable and I wouldn't use it	Count	227	169	4	400
		%	7,9	14,0	5,8	9,6
	I find it unacceptable, but I would use it	Count	26	12	0	38
		%	0,9	1,0	0	0,9
	I find it weird and I wouldn't use it	Count	405	247	6	658
		%	14,0	20,5	8,7	15,8
	I find it weird but I would use it	Count	266	100	1	367
		%	9,2	8,3	1,4	8,8
	I find it acceptable but I wouldn't use it	Count	725	326	9	1060
		%	25,1	27,0	13,0	25,5
	I find it	Count	1235	353	49	1637

	acceptable and I would use it	%	42,8	29,2	71,0	39,4
TOTAL		Count	2884	1207	69	4160
		%	100,0	100,0	100,0	100,0

* p-value: <0.000

Source: The authors.

TABLE 5. Acceptability and Adoptability of **Attitude 5** according to Gender* (in %).

			GENDER			TOTAL
			Feminine	Masculine	Non-binary	
ATTITUDE 5	I find it unacceptable and I wouldn't use it	Count	119	18	5	142
		%	4,1	1,5	7,2	3,4
	I find it unacceptable, but I would use it	Count	127	32	3	162
		%	4,4	2,7	4,3	3,9
	I find it weird and I wouldn't use it	Count	156	26	10	192
		%	5,4	2,2	14,5	4,6
	I find it weird but I would use it	Count	219	55	7	281
		%	7,6	4,6	10,1	6,8
	I find it acceptable but I wouldn't use it	Count	432	139	18	589
		%	15,0	11,5	26,1	14,2
	I find it	Count	1831	937	26	2794

	acceptable and I would use it	%	63,5	77,6	37,7	67,2
TOTAL		Count	2884	1207	69	4160
		%	100,0	100,0	100,0	100,0

* p-value: <0.000

Source: The authors.

TABLE 6. Acceptability and Adoptability of **Attitude 6** according to Gender* (in %).

			GENDER			TOTAL
			Feminine	Masculine	Non-binary	
ATTITUDE 6	I find it unacceptable and I wouldn't use it	Count	31	15	2	48
		%	1,1	1,2	2,9	1,2
	I find it unacceptable, but I would use it	Count	11	4	1	16
		%	0,4	0,3	1,4	0,4
	I find it weird and I wouldn't use it	Count	389	209	17	615
		%	13,5	17,3	24,6	14,8
	I find it weird but I would use it	Count	322	108	8	438
		%	11,2	8,9	11,6	10,5
	I find it acceptable but I wouldn't use it	Count	628	293	23	944
		%	21,8	24,3	33,3	22,7
	I find it	Count	1503	578	18	2099

	acceptable and I would use it	%	52,1	47,9	26,1	50,5
TOTAL		Count	2884	1207	69	4160
		%	100,0	100,0	100,0	100,0

* p-value: <0.000

Source: The authors.
